# Bee-mediated pollination enhances fruit set and seed yield in *Paeonia ostii* ‘Fengdan’: insights into physiological and molecular mechanisms

**DOI:** 10.1093/hr/uhaf224

**Published:** 2025-11-01

**Authors:** Kai-Yue Zhang, Yu-Ying Li, Jun-Yi Bao, Xiang-Nan He, Lin-Feng Chen, Li-Li Guo, Da-Long Guo, Cheng-Wei Song, Chun-Ling He, Xiao-Gai Hou

**Affiliations:** College of Agriculture/Tree Peony, Henan University of Science and Technology, Luoyang, Henan 471023, China; College of Agriculture/Tree Peony, Henan University of Science and Technology, Luoyang, Henan 471023, China; College of Agriculture/Tree Peony, Henan University of Science and Technology, Luoyang, Henan 471023, China; College of Agriculture/Tree Peony, Henan University of Science and Technology, Luoyang, Henan 471023, China; College of Agriculture/Tree Peony, Henan University of Science and Technology, Luoyang, Henan 471023, China; College of Agriculture/Tree Peony, Henan University of Science and Technology, Luoyang, Henan 471023, China; College of Horticulture and Plant Protection, Henan University of Science and Technology, Luoyang, Henan 471023, China; College of Agriculture/Tree Peony, Henan University of Science and Technology, Luoyang, Henan 471023, China; College of Horticulture and Plant Protection, Henan University of Science and Technology, Luoyang, Henan 471023, China; College of Agriculture/Tree Peony, Henan University of Science and Technology, Luoyang, Henan 471023, China

## Abstract

Bee pollination enhances crop productivity and food security globally. However, its impact on pollen performance within pistil tissues and the underlying regulatory mechanisms remain unclear. In this study, artificial self-pollination yielded the highest pollen deposition on stigmas (119879.33 ± 43037.92 grains), followed by bee pollination (95464.60 ± 3985.01 grains). Conversely, bee pollination achieved the highest seed set rate (55.21% ± 1.84%), significantly exceeding the artificial self-pollination rate (7.27% ± 1.87%). A positive correlation was observed between pollen load on the stigmatic pollination band and seed set rate. Bee pollination delivers ample high-quality pollen to the stigmas of oil tree peony, enhancing seed production. Moreover, a trend high correlation was observed between pollen deposition on the stigmatic pollination band and seed set rate. Fluorescence microscopy and endogenous hormone analyses revealed that bee pollination stimulated a rapid increase in ZR, IAA, and GA_3_ levels in the pistil tissues, promoting pollen germination and pollen tube growth. Transcriptome analysis identified *PoFAR2*, a key candidate gene involved in pollen development, in the pistil tissues after bee pollination. This gene exhibits high homology with genes found in other crops. The *PoFAR2* gene localizes to the cell membrane, validating earlier predictions, and exhibits strong transcriptional activity. Silencing *PoFAR2* disrupts pollen development in *Paeonia ostii* ‘Fengdan’ manifesting as structural defects in pollen walls and significantly reduces pollen viability. In conclusion, bees enhance fertilization in oil tree peony by delivering high-quality pollen that promotes germination and pollen tube growth. Crucially, we identified *PoFAR2*, a membrane-localized key gene regulating pollen development. This study establishes a crucial foundation for deciphering the molecular mechanisms by which bee pollination and phytohormone signaling mediate pollen development.

## Introduction

Approximately 75% global major food crop depend on pollinators, with bees, especially *Apis melifera* L., being the most vital [1, 2]. Bee pollination boosts agricultural productivity, food security, and livelihoods by increased fruit, seed, and oil yields while reducing deformities and enhancing commercial grades (fruit color, hardness, shelf life, etc.) [3, 4]. It also improves the quality of fruits and seed oils [5]. Notably, bees continuously and selectively collect pollen during the flowering period, transferring high-quality pollen to receptive stigmas, thereby ensuring efficient pollination [6]. This behavior not only increases pollen deposition on stigmas but also accelerates pollen tube growth and fruit maturation [7, 8]. However, the mechanisms by which bee pollination regulates pollen tube growth and enhances fertilization remain poorly understood.

Pollination is a critical step for flowering plants to complete sexual reproduction [9]. Successful pollination depend on the quantity and quality of pollen, the stigma’s ability to capture and receive pollen, as well as the healthy growth of pollen tubes in the pistil tissue, leading to successful fertilization induction [10–12]. Plant endogenous hormones, along with other signaling molecules, interact in complex ways to regulate cell fate, playing a crucial role in pollen germination, pollen tube growth, and the processes of pollination and fertilization [13]. Studies on plant hybrid compatibility have shown that high levels of ABA in the pistil tissue inhibit pollen tube growth, while elevated levels of endogenous hormones (ZR, IAA, GA_3_) promote pollen-stigma recognition, germination, and fertilization [13, 14]. RNA-seq technology has revealed changes in gene expression profiles in pistil tissues following self-pollination or cross-pollination, identifying genes that regulate pollen germination, pollen tube growth, and fertilization [15–17]. These findings have advanced our understanding of the molecular mechanisms underlying pollen-pistil interactions and pollen tube growth. However, despite numerous studies demonstrating that bees enhance pollen transfer and deposition, thereby improving the quantity and quality of pollen on stigmas [11, 12], there is scarce reporting on the subsequent growth performance, physiological mechanisms, and transcriptional changes of pollen tubes in pistil tissues.

Tree peony (*Paeonia* section *Moutan* DC.) is a perennial deciduous shrub renowned for its large, colorful flowers, which hold significant ornamental, medicinal, and oil value [18, 19]. The seeds of oil tree peony, especially *Paeonia ostii* ‘Fengdan’, widely cultivated in China, are rich in edible oil. The seed oil contains over 90% easily absorbable unsaturated fatty acids, containing over 40% α-linolenic acid, which can improve human suboptimal health [20, 21]. However, oil tree peony flowers are large, pollen-rich, and feature multiple free carpels that develop into aggregated follicles [22]. These flowers lack nectar glands, are partially self-compatible, and exhibit delayed pollen germination, beginning 2 h after self-pollination and reaching the ovary for fertilization after 10 h. In contrast, cross-pollination results in immediate pollen germination upon stigma contact, with double fertilization occurring within 4 to 48 h [20, 23, 24]. Additionally, oil tree peony has a low fruit set rate under self-pollination, relying on pollinating insects to increase fruit set rate [21, 25]. Western honeybees and bumblebees significantly increase seed yield in oil tree peony [26], even mitigating the adverse effects of late spring cold weather [27]. Despite these findings, the impact of bee pollination on pollen germination, pollen tube growth, and plant growth regulators in the pistil tissue of oil tree peony remains unexplored.

In the flowering plant community, bees are better pollen depositors. They optimize pollen collection timing, maximize pollen transfer efficiency, and enhance flower visitation rate and fruit set [12, 28]. Our previous research demonstrated that both western honeybees and bumblebees actively oil tree peony [26]. Notably, the pollen carried by western honeybees exhibits higher vitality compared to that carried by bumblebees or collected artificially, indicating that honeybees selectively gather more viable pollen [29]. Correspondingly, the seed set rate following western honeybees are significantly higher than those from hand pollination, wind pollination, and self-pollination [21]. However, few studies have explored the physiological, biochemical, and transcriptomic changes associated with bee pollination and fertilization that lead to fruit set.

Based on this, the present study used oil tree peony as the research material, with self-pollination as the control, to investigate the superior performance of bee pollination from morphological, physiological, and molecular perspectives. By integrating physiological, biochemical, and RNA sequencing data, we aimed to uncover the physiological and molecular basis for increased seed yield in oil tree peony resulting from bee pollination, offering new perspectives for improving pollination efficiency in this economically important crop. We hypothesize the following: (i) Honeybees enhance seed set by providing a greater quantity of high-quality pollen. (ii) Bee pollination stimulated changes in endogenous hormone levels within the pistil tissue, simultaneously promoting pollen germination and tube growth. (iii) Identification of key candidate genes associated with pollination and fertilization, specifically those involved in pollen development.

## Results

### Improvement of pollen deposition on the stig ma and seed set rate of oil tree peony after bee pollination

**Figure 1:**
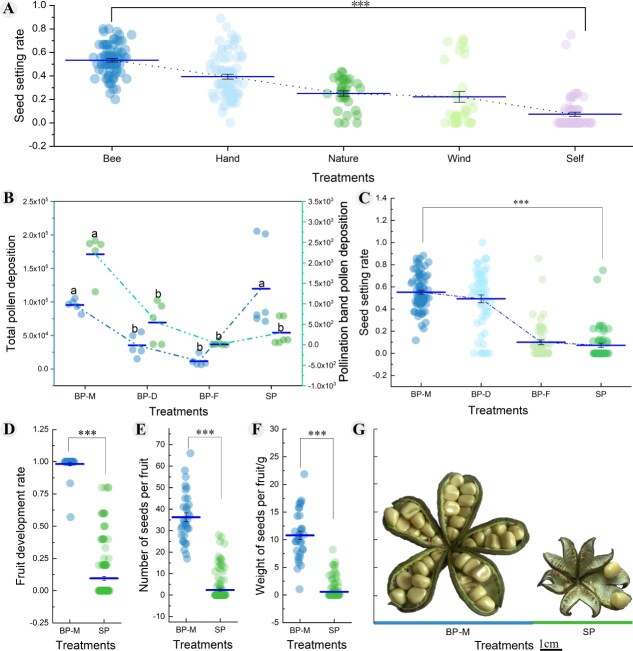
Pollen deposition and fruit set of oil tree peony. A. Seed setting rate under different pollination treatments. B. Number of pollen deposits on stigma. C. Seed setting rate, D. Fruit development rate, E. Number of seeds per fruit, F. Weight of seeds per fruit, G. Fruit set of bee pollination and artificial self-pollination. Bee: Bee pollination, Hand: Hand cross pollination, Nature: Pollination in its natural state, Wind: Wind pollination (non-pollinating insects)，Self: Artificial self-pollination; BP-M: Bees visit multiple times during the flowering period, same as bee pollination, BP-D: Bees visit 1 day, BP-F: Bees visit 1 flower, SP: Artificial self-pollination. Mean ± SD, values followed by different letters were significantly different at *P* < 0.05; ^***^  *P* < 0.01.

Variance analysis of seed set rates in oil tree peony under different pollination treatments revealed that bee pollination resulted in the highest seed set rate (53.33% ± 1.52%), significantly exceeding those of hand pollination, natural pollination, wind pollination (without insect pollinators), and artificial self-pollination (*F* = 69.839, *P* < 0.001). Prior research has documented the impacts of various pollination methods on fruit development, yield, oil extraction efficiency, and seed oil quality [21, 25]. To more precisely identify differentially expressed genes associated with pollination and fertilization, we selected the two treatments exhibiting the most significant phenotypic differences for transcriptome sequencing, based on comprehensive yield data from our preliminary studies (Fig. 1A).

As expected, pollen deposition on stigmas increased with bee visitation frequency (Fig. 1B). Multiple bee visits resulted in total pollen deposition (95464.60 ± 3985.01) was significantly higher than single flower visits (11731.60 ± 3351.23) and 1-day visits (35295.20 ± 7505.05; *F* = 11.589, *P* < 0.01), artificial self-pollination resulted in a pollen deposition of 119879.33 ± 43037.92 grains on the stigma. Additionally, the stigma pollination band after multiple bee visits showed the highest pollen deposition (2211.00 ± 232.44), significantly exceeding one-day visits (544.80 ± 227.43), single flower visits (11.60 ± 5.95), and artificial self-pollination (296.00 ± 206.30, *F* = 27.837, *P* < 0.01).

Correspondingly, a significantly higher seed setting was observed in bee pollination compared to artificial self-pollination (Fig. 1C, *P* < 0.001), with higher bee visitation frequency correlating with greater seed set rates (*F* = 114.711, *P* < 0.001). Furthermore, bee pollination enhanced fruit development rate (98.20% ± 1.38%), number of seeds per fruit (36.24 ± 2.01), and the seed weight per fruit (10.81 ± 0.76 g) in oil tree peony compared to artificial self-pollination (Fig. 1D–G).

### Scanning electron microscopy and fluorescence imaging of the pistil tissues of oil tree peony following bee pollination

**Figure 2:**
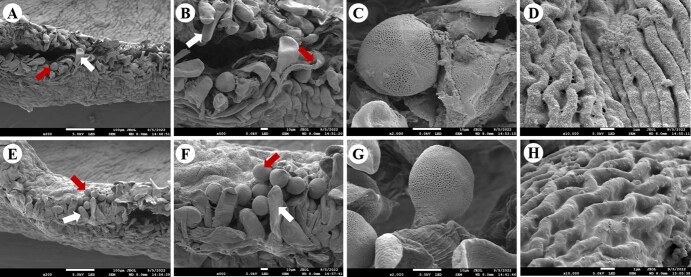
Cytological observations of stigma structures under different pollination treatments. A. Stigmatic pollination band after artificial self-pollination; B, C. Pollen grains attached to the pollination band following artificial self-pollination; D. Stigma surface after artificial self-pollination; E. Stigmatic pollination band after bee pollination; F, G. Pollen grains and papillae structures on the pollination band following bee pollination; H. Stigma surface morphology after bee pollination.

Oil tree peony produces perfect flowers with poorly differentiated styles and stigmas, where the stigma displays a characteristic ‘cockscomb’ morphology formed by two symmetrically fused lobes that curve outward in an ear-like manner to increase the pollination surface area (Fig. 8). A distinct pollination band approximately 1 mm wide (Figs 2A and E and 8B) develops on the stigmatic surface, covered with densely arranged papillae (Fig. 2A, B, E, F, white  arrows) responsible for pollen grain reception. Pollen grains (Fig. 2A, B, E, F, red  arrows) were observed to firmly adhere to the papillae within this pollination band. Comparative analysis revealed no structural differences in papillae between bee pollinated and artificial self-pollinated stigmas, nor any variation in the size or surface ornamentation (fine reticulate pattern) of pollen grains attached to the pollination band (Fig. 2C, G). Similarly, the folded stigmatic surface showed identical morphology under both pollination treatments (Figs 2D, H and 8B).

**Figure 3:**
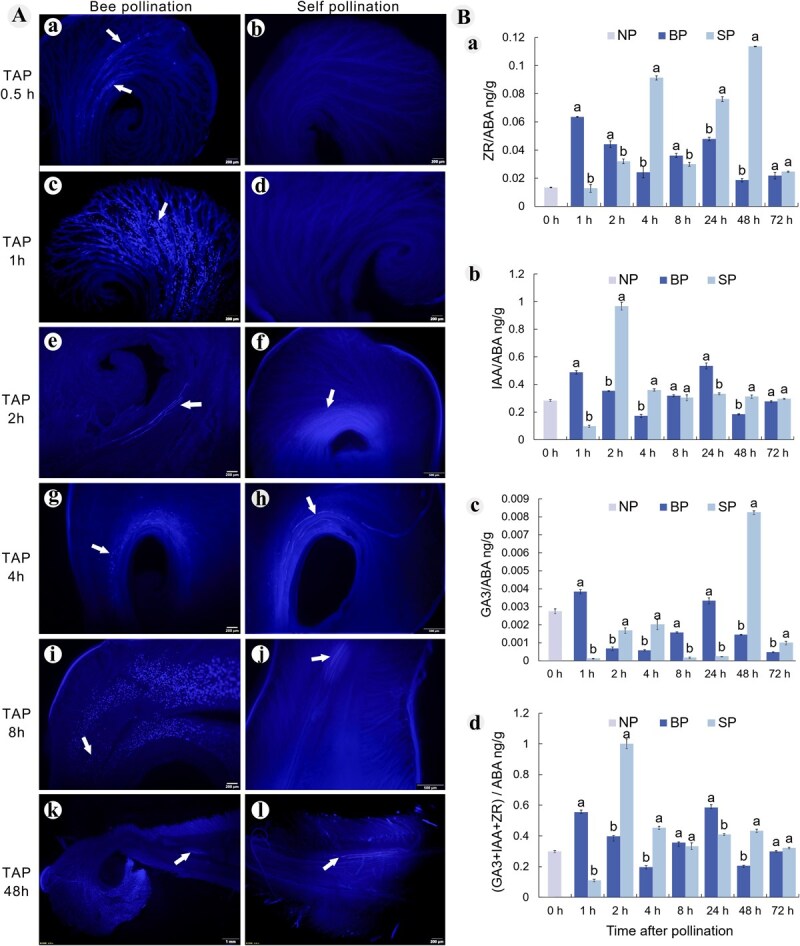
A. Fluorescence observation of pollen tube growth under different pollination treatments. a-b. TAP 0.5 h, after bee pollination, a small amount of pollen germination occurs, while no pollen germination was observed in artificial self-pollination. c, d. TAP 1 h, abundant pollen tubes observed on the stigma after bee pollination, while no pollen germination was observed in artificial self-pollination. e, g, i. TAP 2 h, TAP 4 h, TAP 8 h, after bee pollination, abundant pollen tubes grew towards the style and penetrated the style. f. Pollen tubes observed on the stigma 2 h after artificial self-pollination; h, j. TAP 4 h, TAP 8 h, after artificial self-pollination, pollen tubes grew towards the style. k. Abundant pollen tubes entered the ovary 48 h after bee pollination. l. A small number of pollen tubes entered the ovary 48 h after artificial self-pollination. White arrows indicate observed pollen tubes. TAP: Time after pollination. Our imaging strategy prioritized capturing entire pistil structures to comprehensively document pollen tube positioning within the developmental context. B. Dynamic changes in protective enzyme activity and endogenous hormone levels in the pistil tissue after different pollination treatments. a. Dynamic changes of ZR/ABA, b. Dynamic changes of IAA/ABA, c. Dynamic changes of GA_3_/ABA, d. Dynamic changes of (IAA + ZR + GA_3_)/ABA. NP: Non pollination, BP: Bee pollination, SP: Artificial self-pollination. Mean ± SD, values followed by different letters were significantly different at *P* < 0.05.

Fluorescence microscope revealed that pollen germination occurred on the stigma 0.5 h after bee pollination, while no pollen germination was observed after artificial self-pollination (Fig. 3A-a, b). By 1 h, numerous pollen grains had germinated on bee pollination stigmas, whereas elongated pollen tubes were rare in self-pollinated stigmas (Fig. 3A-c, d). At 2 h, many pollen tubes from bee pollination had penetrated the style, while elongating pollen tubes began to appear in artificial self-pollinated stigmas, with bee pollination showing a greater number of pollen tubes (Fig. 3A-e, f). Between 4 and 8 h post-pollination, a significant number of pollen grains passed through the style into the ovary, with more pollen tubes from bee pollination penetrating the style (Fig. 3A-g–j ). By 48 h, numerous pollen grains were still germinating and elongating toward the style, and a substantial number of pollen tubes had entered the ovary, with bee pollination again showing more extensive pollen tubes growth (Fig. 3A-k, l).

### Changes in endogenous hormone ratios in the pistil tissues of oil tree peony at different time points post-pollination

Based on fluorescence microscopy of pistil tissues, we analyzed temporal variations in endogenous hormone ratios at different post-pollination intervals. After bee pollination, the trends in endogenous hormone ratios were consistent, showed two peaks (BP_1_ and BP_24_). The ratios ZR/ABA (BP_1_: 0.06 ± 0.002 ng/g), IAA/ABA (BP_1_: 0.49 ± 0.01 ng/g), GA_3_/ABA (BP_1_: 0.004 ± 0.0001 ng/g), and (IAA + ZR + GA_3_)/ABA (BP_1_: 0.55 ± 0.01 ng/g) all peaked at 1 h post-pollination. In contrast, artificial self-pollination resulted in different trends: ZR/ABA showed two peaks (SP_4_: 0.09 ± 0.002 ng/g and SP_48_: 0.11 ± 0.001 ng/g), while IAA/ABA (SP_2_: 0.97 ± 0.02 ng/g), GA_3_/ABA (SP_48_: 0.08 ± 0.001 ng/g), and (IAA + ZR + GA_3_)/ABA (SP_2_: 1.001 ± 0.02 ng/g) exhibited only one peak. Compared to NP_0_, endogenous hormone ratio increased within 1 h after bee pollination but decreased initially after artificial self-pollination, only rising after 2 h (Fig. 3B-a–d).

### Global analyses of transcriptome data from pollinated pistil of oil tree peony

We collected 45 cDNA libraries from pistil tissues of oil tree peony subjected to self-pollination, bee pollination, and non-pollinated controls. After quality screening, a total of 305.72 Gb of clean data was obtained. With each sample yield ≥5.86 Gb, a GC content >44.30%, and a Q30 base percentage ≥ 92.38% (Table S1). Using the tree peony genome (https://ftp.cngb.org/pub/CNSA/data5/CNA0050666), reads from each sample aligned to the reference genome with an efficiency 79.17% to 85.75% (Table S2). Based on the alignment results, we performed alternative splicing prediction and gene structure optimization, identifying 68 880 genes for further analysis. Compared to the NP group, 1558 differentially expressed genes (DEGs) were identified in the BP group, showing significant enrichment in the terpenoid metabolic process (GO:0006722) and MAPK signaling pathway (ko04010), among others (Fig. S1A and B). Analysis of DEGs between the SP and NP groups revealed 2831 DEGs, primarily enriched in the secondary metabolic process (GO:0019748) and protein processing in the endoplasmic reticulum (ko04141), among others. Additionally, 23 DEGs were enriched in the regulation of pollen tube growth (GO:0080092) (Fig. S1C and D).

**Figure 4:**
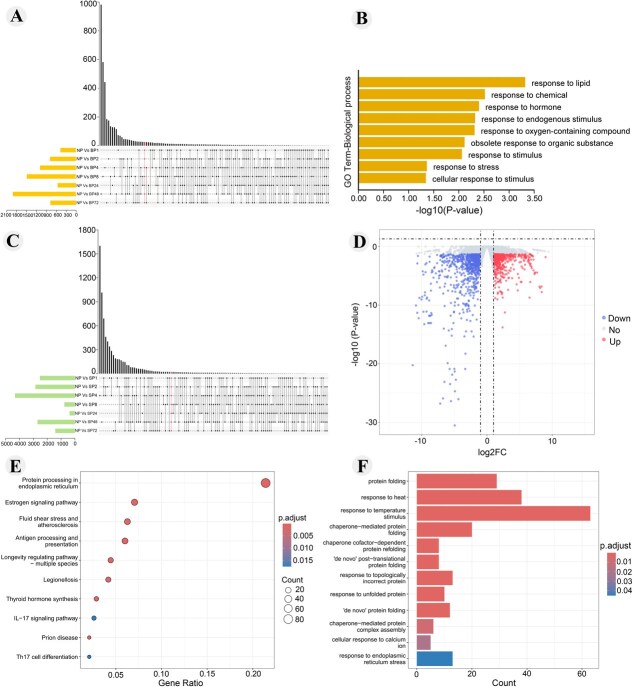
Differential gene expression analysis after bee and artificial self-pollination. A. Venn diagram of DEGs in BP, B. GO analysis of DEGs in BP, C. Venn diagram of DEGs in SP, D. Differentially expressed gene analysis at 2 h post-pollination, E. GO analysis of DEGs in BP_2_ vs SP_2,_ F. KEGG analysis of DEGs in BP_2_ vs SP_2_.

To further investigate the DEGs involved in pollination and fertilization in of oil tree peony, 26 intersecting DEGs were identified during bee pollination (Fig. 4A). These intersecting DEGs were significantly enriched in responses to lipid (GO:0033993), chemical (GO:0042221), and hormone (GO:0009725) (Fig. 4B). In contrast, 21 intersecting DEGs were identified during artificial self-pollination (Fig. 4C). but these DEGs lacked significant GO and KEGG enrichment pathways.

Fluorescence microscopy revealed that 2 h post-pollination is a critical period for pollination and fertilization in oil tree peony. At this stage, pollen tube in bee pollinated flowers began to penetrating the style, while pollen on the stigma of artificial self-pollinated flowers was germinating and elongating. Bee pollinated flowers exhibited more extensive pollen tube growth compared to artificial self-pollinated ones. Compared to the SP_2_ group, 1407 DEGs were identified in the BP_2_ group, including 600 up-regulated genes and 807 down-regulated genes (Fig. 4D). These DEGs were significantly enriched in protein processing in the endoplasmic reticulum (ko04141), the estrogen signaling pathway (ko04915) and were majority associated with the response to temperature stimulus (GO:0009266) (Fig. 4E and F).

### Analysis of differential gene expression trend

**Figure 5:**
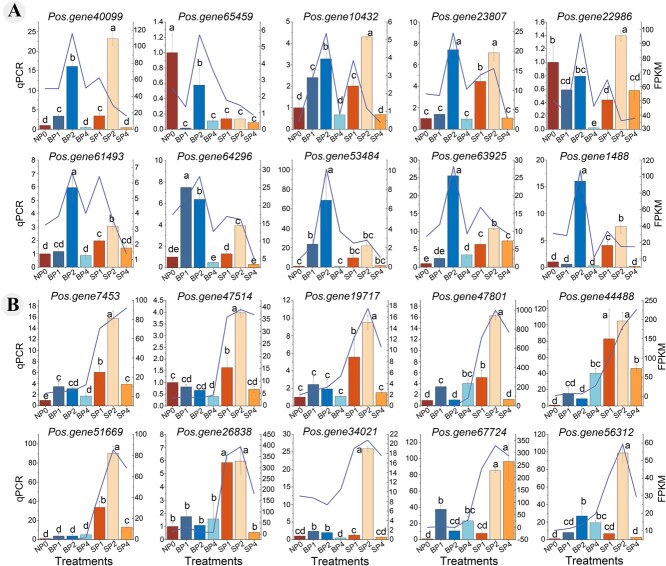
Quantitative real-time PCR analysis of gene expression after different pollination treatments. A. The top 10 genes with relatively high expression levels in BP_2_, B. The top 10 genes with relatively high expression levels in SP_2_. Mean ± SD, *n* = 3. Values followed by different letters were significantly different at *P* < 0.05.

Cluster, expression trend and GO enrichment analysis were performed for DEGs in the BP group. Based on their expression patterns, the DEGs were divided into 8 clusters, each containing 1905 to 2750 specific DEGs. Among these, 2081 DEGs were significantly up-regulated in BP_1_ and grouped into Cluster 8; 1095 DEGs were significantly up-regulated in BP_2_ and assigned to Cluster 4; 1915 DEGs and 2667 DEGs were highly expressed in BP_4_, clustering in Cluster 3 and Cluster 5, respectively; 2750 DEGs were highly expressed in BP_8_ and clustered in Cluster 1; 2253 DEGs were highly expressed in BP_24_ and clustered in Cluster 2; 1949 DEGs were highly expressed in BP_48_ and clustered in Cluster 6; 2560 DEGs were highly expressed in BP_72_ and clustered in Cluster 7 (Fig. 5A).

The integration of early hormone levels and pollen tube elongation patterns confirmed that 2 h post-pollination was a critical period for pollination and fertilization in oil tree peony. We therefore focused on genes expressed at this time point. By analyzing the expression trends of DEGs within each Cluster, we found that DEGs in Cluster 4 showed a significant increased at BP_2_. These DEGs were primarily enriched in the following biological processes: xylan metabolic process (GO:0045491), plant-type secondary cell wall biogenesis (GO:0009834), plant-type cell wall biogenesis (GO:0009832), phenylpropanoid metabolic process (GO:0009698), fatty acid derivative metabolic process (GO:1901568), lipid metabolic process (GO:0046486), and fatty-acyl-CoA metabolic process (GO:0035336) (Fig. 5B).

### Validation of transcriptome data and expression patterns

**Figure 6:**
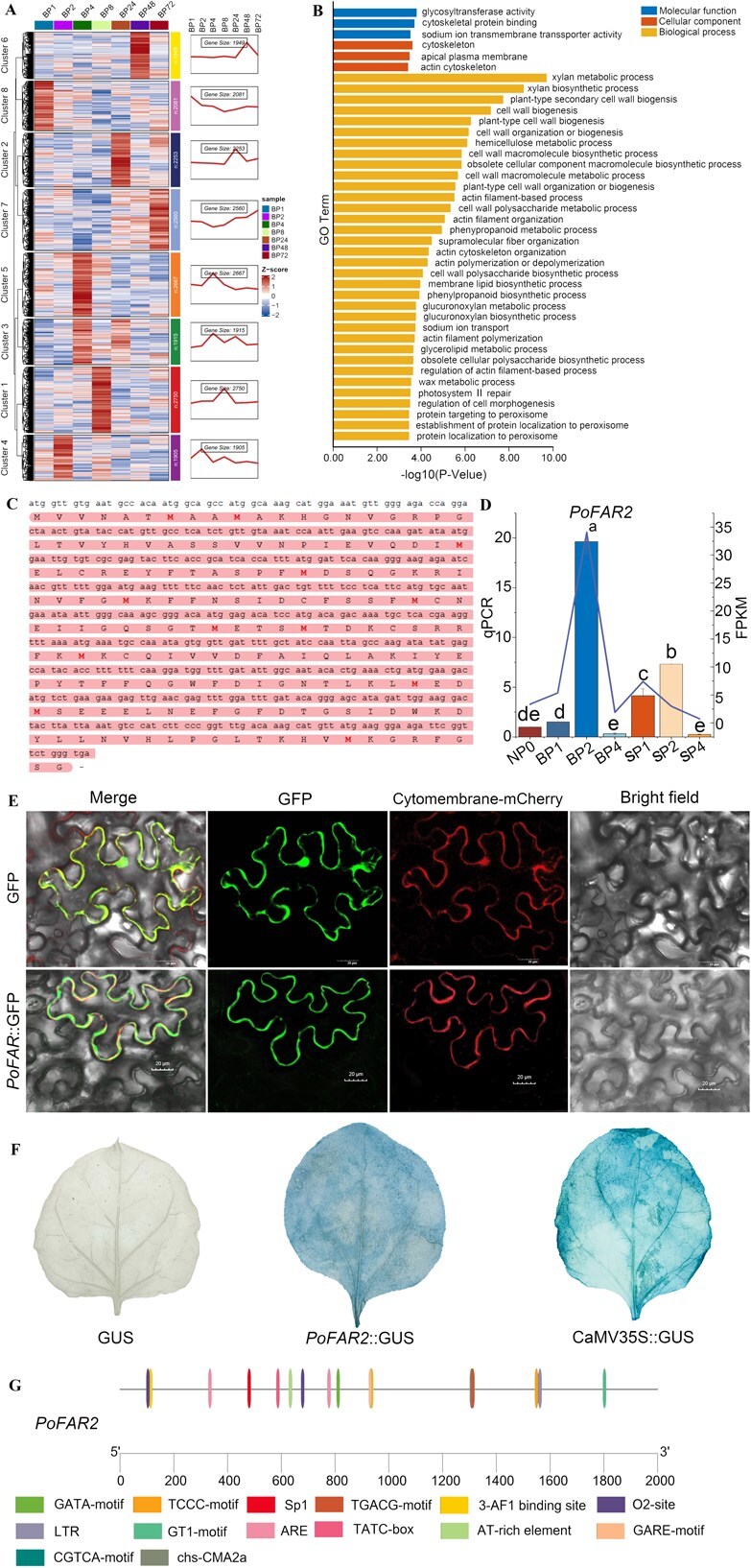
Identification of *PoFAR2*, a key candidate gene for pollination in honeybees. A. Analysis of the expression trend of DEGs in BP group. B. GO analysis of cluster 4 genes. C. *PoFAR2* sequence information. D. *PoFAR2* expression, Mean ± SD, values followed by different letters were significantly different at *P* < 0.05. E. Subcellular localization. F. *PoFAR2* promoter activity. G. *PoFAR2* promoter sequence analysis.

At 2 h post-pollination, 21 highly expressed DEGs potentially involved in pollination and fertilization were identified for fluorescence quantitative analysis (Figs 5 and 6D). The developmental stages of bee pollination and artificial self-pollination at 0, 1, 2 and 4 h were determined based on physiological indexes and fluorescence microscopy images. The qRT-PCR results showed relative expression levels consistent with RNA-Seq trends, confirming the reliability of the transcriptome sequencing data.

It is worth noting that we focus on Pos.gene.78836 in cluster 4 (Fig. 6A and B), a gene homologous to *FAR2* (Fatty Acyl-CoA Reductase 2) in *Arabidopsis thaliana*, which is essential for pollen exine patterning by mediating pollen wall formation [30]. This gene was cloned from oil tree peony pistil tissue after bee pollination and named *PoFAR2*. The 549-bp sequence of *PoFAR2* was shown in Fig. 6C. Its expression in pistil tissues increased initially and then decrease at 0, 1, 2, 4 h after bee pollination, with significant high expression observed at BP_2_ (Fig. 6D).

To explore *PoFAR2* function, we constructed a 35S–*PoFAR2*–GFP fusion expression vector, using a 35S–GFP empty vector as a negative control. Fluorescence signal from 35S-*PoFAR2*-GFP was localized to the cell membrane, consistent with the predicted membrane localization, while GFP signals in the negative control were observed in both the nucleus and cell membrane (Fig. 6E).

Meanwhile, we also assessed *PoFAR2* promoter activity by infiltrating tobacco leaves with *Agrobacterium* carrying the *PoFAR2* promoter. A GUS vector with the 35S promoter served as the positive, and a promoterless GUS vector was the negative control. Leaves with the *PoFAR2* promoter showed lighter blue staining than the 35S-positive control, which may indicate that the *PoFAR2* promoter was active (Fig. 6F). Analysis of the 2000-bp upstream region of *PoFAR2* identified multiple cis-acting elements, including six light-responsive elements (GATA-motif, 3-AF1 binding site, Sp1, TCCC-motif, chs-CMA2a, GT1-motif), two gibberellin-responsive elements (TATC-box), two MeJA-responsive elements (CGTCA-motif), two anaerobic induction elements (ARE), two zein metabolism regulation elements (O_2_-site), one low-temperature responsiveness element (LTR), and an AT-rich DNA binding protein (ATBP-1) site (AT-rich element) (Fig. 6G).

### Silencing *PoFAR2* disrupted pollen wall development in tree peony

**Figure 7:**
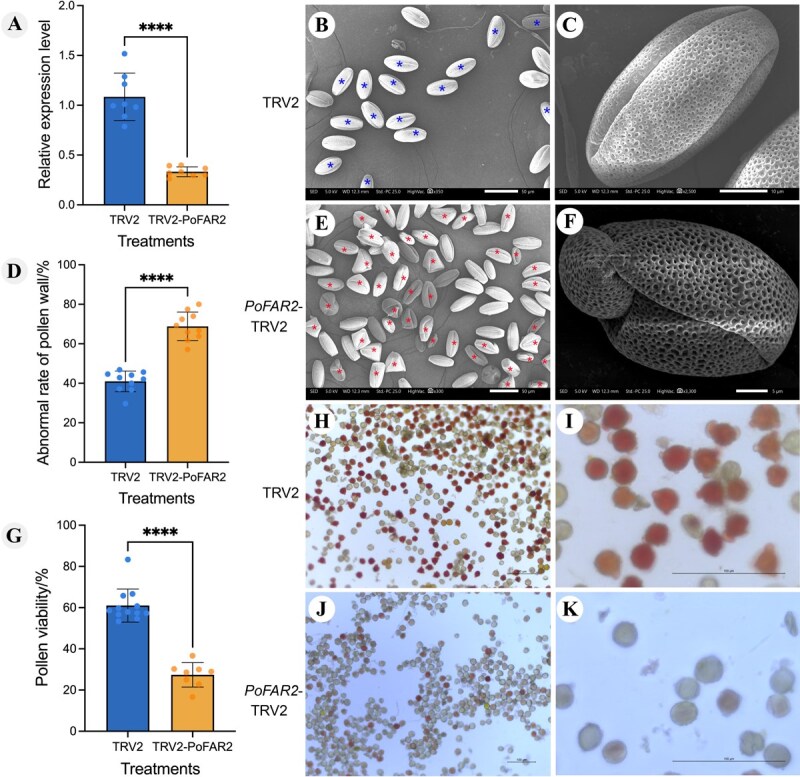
Silencing *PoFAR2* results in pollen development abnormal in *P. ostii*. A. Expression of *PoFAR2* in TRV2 and *PoFAR2* silenced lines. B, C. Morphology of normal mature pollen grains; asterisks indicate normally developed pollen. Scale bars = 50 μm (B), 100 μm (C). E, F. Abnormal mature pollen morphology; asterisks mark abnormally developed pollen grains. Scale bars = 50 μm (D), 5 μm (E). D. Abnormal rate of pollen for TRV2 and *PoFAR2*-TRV2. G. Pollen viability in TRV2 and *PoFAR2*-TRV2. H-K. Vitality of mature pollen in *P. ostii* ‘Fengdan’ for TRV2 and *PoFAR2*-TRV2, respectively. Dark red indicates high pollen vitality, while light red or lack of color indicates low or no vitality. Scale bar = 100 μm. Mean ± SD, ^****^  *P* < 0.01.

Using virus-induced gene silencing (VIGS), we suppressed *PoFAR2* expression in *P. ostii* ‘Fengdan’ (Fig. 7A). Scanning electron microscopy (SEM) revealed stark morphological differences. Control pollen showed 60% ± 1.65% normal development. These grains were prolate or spherical with tricolporoidate apertures (widest at mid-groove, narrowing toward ends) and featured reticulate exine ornamentation with variably sized circular/ovoid lumina (Fig. 7B and C). *PoFAR2*-silenced pollen exhibited 68.84% ± 2.27% abnormality. Grains displayed malformed corporate, irregular contours, and abnormal curling/collapse (Fig. 7D–F).

To determine if these defects caused pollen abortion, we performed TTC (2,3,5-triphenyltetrazolium chloride) staining. Controls showed high viability: 61.01% ± 2.33% stained deep red (Fig. 7G–I). Conversely, only 27.36% ± 2.30% of *PoFAR2*-silenced pollen stained light red, with rare red grains (Fig. 7G, J–K). The unstained proportion matched the developmental abnormality rate. Thus, *PoFAR2* silencing impairs pollen wall formation and severely compromises pollen viability.

## Discussion

### Bee pollination improves pollen quality in oil tree peony stigma

Bee pollination is crucial for seed production in oil tree peony, significantly increasing fruit set rates. We have uncovered the mechanisms underlying by which bee pollination enhances productivity. Successful pollination and fertilization depend on the stigma receiving sufficient high-quality pollen during its receptive period [10, 31]. Bees preferentially visit oil tree peony, efficiently transferring robust pollen to receptive stigmas and ensuring effective pollination [29].

Bees preferentially visit oil tree peony flowers, selectively collecting high-viability pollen, which surpasses manually collected pollen in quality [29]. This suggests that pollen source influences pollination efficacy. Similar to hand pollination, bees frequently contact stigmas during pollination, and their superior efficacy warrants further investigation. Excessive bumblebee activity, as seen in raspberries, can harm stigmas, impeding pollen deposition and ovule fertilization [32]. Pollinator behavior significantly affects pollen transfer and deposition; longer bees visit increase pollen deposition, enhancing seed number and fruit quality [33]. However, raspberry studies show fruit set does not linearly correlate with bee visit frequency [32]. Our findings confirm that increased bee–stigma interactions elevate pollen deposition, but the stigma’s pollen acceptance threshold remains to be determined.

Pollen deposition on stigmas, rather than pollinator visitation, better predict crop yield [34]. A bee's grooming behavior during continuous flower visits enhances pollen carrying capacity, increasing the amount and diversity of pollen on stigmas, which promotes thorough fertilization, higher seed numbers and improved fruit quality [33, 35, 36]. In oil tree peony, increased bee visits elevate pollen deposition and seed production. However, the relationship between visitation frequency and fruit set rate is nonlinear; excessive visits over-deposit pollen, causing pollen grains or pollen tube interference, reduced fertilization and lower fruit production [32, 37]. The optimal bee visitation for oil tree peony and its impact on pollen deposition and fruit set require further study. Garibaldi et al. [34] reported that honeybee visits increase pollen deposition more than fruit set, likely due to high quantities of poor-quality (i.e. self) pollen. While in oil tree peony, artificial self-pollination also results in high pollen deposition, its low seed set rate reflects poor pollen quality. Our findings highlight the importance of managed bees in providing high-quality pollen to enhance seed production.

### Bee pollination promotes more pollen germination and pollen tube growth

The amount of pollen deposition on stigmas is closely related to the physiological processes, including pollen tube growth and fruit set. Higher pollen deposition increases the probability of successful fertilization in plants and promotes the early development of the plant’s ovary [38]. In our study, bee pollination resulted in lower pollen deposition on stigmas compared to artificial self-pollination but achieved higher pollen germination rates and faster pollen tube growth. This can be attributed to several factors. First, oil tree peony, a partially self-compatible cross-pollinated plant, benefits from the high-quality pollen transferred by bees [21]. Second, increased exogenous pollen deposition stimulates more pollen tubes in the style, and their competition accelerates pollen tubes growth [7]. Third, multiple pollinator visits often initiate pollen tube growth capable of ovule fertilization, enhancing fruit and seed production [8, 39]. Conversely, incompatible pollination inhibit pollen tube growth, and irregular callose deposition may block pollen tubes entirely [40].

### Bee pollination promotes the quicker attainment peak of endogenous hormones ratios

Pollination, as an exogenous stimulus, induces changes in enzyme activity and hormone levels in pistil tissues, closely linked to pollen germination and pollen tube growth [41, 42]. Specific concentrations of IAA and GA_3_ promote in vitro pollen germination and pollen tube growth, while ABA inhibits pollen germination but a certain promoting effect on pollen tube growth [43]. In hybrid pollination studies, high levels of IAA, GA_3_, ABA, and ZR support pollen tube growth, with balanced ratios [e.g. GA_3_/ABA, IAA/ABA, IAA/ZR, ZR/ABA, and (GA_3_ + ZR + IAA)/ABA] reflecting pollination affinity [13, 42, 44]. Fluorescence observations showed rapid pollen germination and pollen tube growth 1 h after bee pollination, whereas artificial self-pollination delayed these processes by 2 h. Compared to the nonpollination controls (0 h), endogenous hormones ratios increased 1 h after bee pollination and surpassed those in artificial self-pollination, indicating that IAA, GA_3_, ABA, and ZR collectively promote pollen germination and tube growth. In contrast, artificial self-pollination initially reduced hormone ratios, which began to rise 2 h post-pollination, while ABA suppressed pollen germination. These finding highlight the importance of balanced hormone ratios in regulating pollen germination and pollen tube growth, and indirectly show that bee pollination delivers high-affinity pollen to oil tree peony stigmas, stimulating endogenous growth regulators and enhancing pollen germination and pollen tube growth.

### Identification of key candidate genes associated with pollen development

Pollination and fertilization process involve multigene regulatory networks. In this study, we analyzed gene expression in oil tree peony during bee pollination and artificial self-pollination, identifying 68 880 genes. Among these, 21 and 26 intersecting DEGs in were unique to artificial self-pollination and bee pollination, respectively. Bee pollination DEGs were mainly enriched in pathways such as the plant hormone signal transduction (ko04075), the circadian rhythm-plant (ko04712), the protein processing in endoplasmic reticulum (ko04141), and the endocytosis (ko04144), consistent with findings in sweet cherry pollen tube lncRNAs [15]. The 1- to 6-h after-pollination window is critical for pollen tube development, inter-variety pollen tube competition, and stigma changes [45, 46]. Focusing on 2 h after pollination (Fig. S3), we identified *PoFAR2* in cluster 4, a gene of interest involved in the following pathways: lipid metabolic process (GO:0006629), Cellular Component: integral component of membrane (GO:0016021), transmembrane transporter activity (GO:0022857), fatty-acyl-CoA reductase (alcohol-forming) activity (GO:0080019), alcohol-forming fatty acyl-CoA reductase (ko13356), and cutin, suberine and wax biosynthesis (ko00073).

Following *PoFAR2* silencing, pollen grains exhibited malformed apertures, irregular contours, and abnormal curling/collapse. Notably, functional conservation was observed in Gossypium hirsutum, where CRISPR/Cas9-mediated knockout of *GhFAR2* resulted in complete male sterility—manifested as pollenless anthers with abnormal atrophy further confirming FAR2’s essential role in pollen development across eudicots [47]. *PoFAR2* is highly homologous to *FAR2* in *Arabidopsis thaliana*, where *AtMS2/FAR2* is essential for spore (pollen) outer wall development [48]. The pollen wall, a dense layer surrounding the male gametophyte, is critical for pollen grain integrity. During another development, pollen wall deposition in pollen grains is one of the most critical events. Its absence can hinder pollen development, leading to pollen degradation [49]. *PoFAR2* is annotated as having fatty-acyl-CoA reductase (alcohol-forming) activity, and fatty acyl-CoA participates is also crucial for pollenin precursors. Fatty acyl-CoA synthetase is essential for pollen development and pollenin biosynthesis [50]. *OsDPW* encodes a fatty acyl-CoA reductase with high homology to the Arabidopsis *MS2* gene. This gene participates in nutrient synthesis within rice anther tapetum cells. Mutations in *OsDPW* cause increased plastids in tapetum, reduced Ubisch body production, abnormal pollen exine development, and microspore degeneration [51]. In the current study, *PoFAR2* silencing similarly resulted in abnormal pollen development in *P. ostii* ‘Fengdan’. The gene’s role in ‘Fengdan’ pollen development requires further mechanistic investigation.

The predicted and experimentally confirmed subcellular localization of *PoFAR2* to the cell membrane suggests its potential role in membrane-associated processes, such as lipid metabolism or signal transduction [52]. There have also been studies reported other pollen development-associated *FARs,* such as including *AtMS2/FAR* [48], *OsDPW* [53], and *ZmMs25* [54]*,* localize to the plastid envelope. Analysis of the *PoFAR2* promoter revealed gibberellin-responsive elements (TATC-box), consistent with the role of GA_3_ in pollination and fertilization. As well as MeJA-responsiveness elements (CGTCA-motif). These findings suggest that *PoFAR2* may integrate gibberellin and jasmonic acid signaling pathways. Jasmonic acid (JA) mediates plant responses to temperature stress, and disruptions in JA synthesis can lead to abnormal pollen development and anther indehiscence [55]. Additionally, light-responsive elements (e.g. GATA-motif) in the promoter imply that *PoFAR2* could integrate environmental cues, such as light, into cellular responses. Together, these findings suggest that *PoFAR2* functions at the cell membrane to mediate hormone and environmental signal transduction during pollen development. Future studies should focus on validating the regulatory effects of these signals on *PoFAR2* expression and elucidating its specific roles in membrane-related processes. However, it is important to note that functional validation of *PoFAR2* is currently limited by technical challenges in the genetic transformation of *P. ostii*, a perennial woody plant with long regeneration cycles. Despite these limitations, we are actively developing virus-induced gene silencing (VIGS) protocols for transient validation in stigma, which will provide further insights into the functional roles of *PoFAR2* in pollination and fertilization.

In addition, the gene Pos.gene10432, identified in BP_2_, is highly homologous to *Arabidopsis GLIP2*, which regulate IAA expression [56] and promotes pollen tube growth [57]. Pos.gene53484 exhibits high homology with the *Arabidopsis FLS1*, encoding flavonol synthase that catalyze the flavonol production from dihydroflavonol [58]. Flavonols maintain pollen tube growth and integrity by regulating ROS homeostasis under high-temperature stress, a process critical for pollen germination [59]. Pos.gene63925 is homologous to *Arabidopsis WSD1*, a wax synthase/acyltransferase involved in wax synthesis [60]. Plant wax, a hydrophobic layer on pistil surfaces, facilitates pollen-stigma signal recognition [61, 62]. The specific functions of these genes require further investigation. In SP_2_, Pos.gene56312 is homologous to *Arabidopsis ABI5*, a gene involved in ABA-regulated expression [63]. Since ABA inhibits pollen tube in pistil [13], further research and verification are needed to determine whether Pos.gene56312 regulates pollen tube growth in oil tree peony.

## Conclusion

This study established that bee pollination enhances pollination efficiency by providing sufficient high-quality pollen, leading to increased seed set. It promotes greater pollen germination and tube growth on stigmas while stimulating more effective action of endogenous hormones in the pistil tissue. Notably, this work integrates bee pollination technology with transcriptome sequencing in oil tree peony for the first time. Through differential gene expression analysis, we identified *PoFAR2* as a key candidate gene associated with pollen development, localized to the cell membrane and exhibiting strong promoter activity. Its expression is likely regulated by GA_3_ and jasmonic acid signaling. Silencing *PoFAR2* disrupts pollen development (e.g. structural defects in pollen walls) and reduces pollen viability in *P. ostii* ‘Fengdan’. Although the precise regulatory mechanisms of *PoFAR2* require further investigation, our findings reveal a broader signaling network involving bee pollination, phytohormones, and pollen maturation. Elucidating the tripartite interactions among bees, hormones (notably GA_3_ and jasmonic acid), and pollen development is essential. This study provides critical theoretical insights for enhancing oil tree peony breeding efficiency and advancing ecological management strategies for crop pollination systems.

## Material and methods

### Pollination treatment

Experiment was conducted during the flowering season of peonies in April 2022 at the planting base of *Paeonia ostii* ‘Fengdan’ in Donghua Mudanyuan, Yibin District, Luoyang City (N 34°38′30″, E 112°39′43″, altitude 125.5 m). A controlled net room [45 m (length) × 8 m (width) × 3.2 m (height)] was used, and western honey bees (*Apis mellifera* L.; Hereinafter referred to as Bee) were introduced, and a same blank control was established. Five different pollination treatment methods were set up: bee pollination (Bee), Hand pollination (Hand), Nature pollination (Nature), Wind pollination (Wind), Artificial self-pollination (Self). The methodologies for hand pollination, wind pollination, and nature pollination are described in detail in Zhang et al. [21, 25].

Bee Pollination (Bee, BP-M): In the controlled net room supplemented with bees, tree peony flowers were visited multiple times by bees throughout the flowering period. The statistical period was defined as the entire flowering duration. To further evaluate pollination efficiency, two additional treatments were established: Bees Visit 1 Day (BP-D): Bees were allowed to visit flowers multiple times from 8:00 a.m. to 6:00 p.m., with the statistical period defined as one day. Bees Visit 1 Flower (BP-F): A single bee was allowed to visit one flower, from landing to departure, with the statistical period defined as the duration of that visit.

Artificial self-pollination (Self, SP): In the control net room, flower buds were bagged with sulfuric acid paper to allow only self-pollination, excluding pollinators and wind (Self). To ensure accurate sampling and confirm stigma receptivity, flower stems were gently agitated to facilitate pollen transfer onto the stigma, a procedure hereafter referred to as artificial self-pollination (SP).

**Figure 8:**
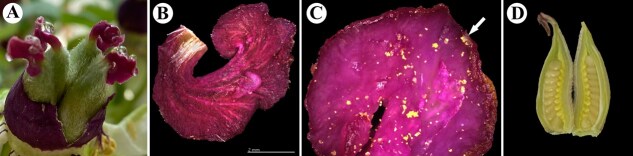
Pistil tissue of oil tree peony. A. Pistil, B. Stigma, C. Pollination band, D. Ovary RNA extraction and library preparation.

Non-pollinated (NP): Stigmas were emasculated before anther dehiscence to prevent pollen reception, ensuring no pollination occurred.

### Sample collection and testing methods

(1) Fruit set investigation

Before flowering, 100 tree peony plants were randomly selected in the controlled net room, and bees were introduced. From each plant, two flower buds at the same flowering stage were chosen: one for bee pollination and the other bagged for self-pollination until the end of flowering. After fruits maturation, the fruit development rate (number of follicles with seeds/total number of follicles), seed setting rate (number of developed seeds per follicle/total number of ovules per follicle), number of seeds per fruit, and weight of seeds per fruit were recorded, following the methods described by Zhang et al. [21].

(2) Stigma pollen deposition and pollen viability

Before flowering, 40 tree peony flower buds at the same developmental stage were bagged. On the third day of flowering, the bags were removed, and the buds were divided into four groups: BP-M group, BP-D group, BP-F group, and SP group. Stigmas were sampled 10 h post-pollination (Fig. 8A) and transported to the laboratory in centrifuge tubes. They were washed with sodium pyrophosphate, counted using a hemocytometer, and sectioned for observation under an Olympus optical microscope. Pollen viability was assessed using TTC (2,3,5-triphenyltetrazolium chloride) staining [24].

(3) Cytological structure observation, physiological and biochemical indicators, and transcriptome sequencing samples

One hundred tree peony flowers at the same developmental stage were randomly selected and bagged. After bee pollination (BP_1_, BP_2_, BP_4_, BP_8_, BP_24_, BP_48_, BP_72_) and artificial self-pollination (SP_1_, SP_2_, SP_4_, SP_8_, SP_24_, SP_48_, SP_72_), stigmas tissues were sampled at 1, 2, 4, 8, 24, 48, and 72 h (Fig. 8A). Non-pollinated (NP_0_), i.e. 0 h, were also sampled. There were for each treatment and time period point, six biological replicates divided into two groups (three replicates each). One group was fixed in FAA solution (ethanol: glacial acetic acid: formalin = 18:1:1) for over 48 h and stored at 4°C, while the other was snap-frozen in liquid nitrogen and stored at −80°C. Additionally, three replicates of stigmas from continuous bee pollination and artificial self-pollination were fixed in glutaraldehyde for electron microscopy and stored at 4°C (Fig. 8B).

The morphological structure of stigmas was observed using scanning electron microscopy, following He *et al.* [64]. Fluorescence microscopy was performed using the benzidine blue staining method described by Chen *et al.* [65]. cis-Zeatin Riboside (ZR), indole-3-acetic acid (IAA), gibberellin A_3_ (GA_3_), and abscisic acid (ABA) Endogenous hormone levels were quantified using reagent kits and an Agilent 1290 HPLC-MS/MS system coupled with an AB Sciex Qtrap 6500 mass spectrometer (Nanjing Ruiyuan Biotechnology Co., Ltd).

Forty-five pistil tissue samples from different pollination treatments and time points were sent to Biomarker Technologies in Beijing for transcriptome sequencing. Total RNA was extracted using the RNAprep Pure Plant Kit (Tiangen, Beijing, China) following the manufacturer’s instructions. For each sample, 1 μg of RNA was used to prepare sequencing libraries with the Hieff NGS Ultima Dual-mode mRNA Library Prep Kit for Illumina (Yeasen Biotechnology, Shanghai, China). Index codes were added to assign sequences to individual samples, and library fragments were purified using the AMPure XP system (Beckman Coulter, Beverly, USA). Libraries were sequenced on an Illumina NovaSeq platform to generate 150-bp paired-end reads. Adaptor sequences and low-quality sequences were removed to obtain clean reads, which were then mapped to the reference genome [66]. Differential expression analysis between groups was performed using the DESeq2, with adjusted *P* values calculated using the Benjamini-Hochberg method to control the false discovery rate. Genes with an adjusted *P*-value <0.01 and a fold change >2 were identified as differentially expressed.

### Analysis of expression trend of differentially expressed genes

Gene modules with highly consistent expression patterns were identified from BP group DEGs using clusterGVsi package in R v4.4.1. Genes with low FPKM ([FPKM] < 1) were filtered out, resulting in 18 080 genes for further analysis. Expression trends were fitted using the Mfuzz method.

To validate the RNA-Seq data, qRT-PCR was performed to measure the expression levels of hub genes, with *EF1-α* as the reference control [67]. Primers were designed using Primer Premier 5.0, and sequences are listed in Table S3. All samples were analyzed in triplicate, and relative expression levels were calculated using the 2^−ΔΔC_T_^ method [68].

### Gene cloning

Total RNA from the BP_2_ was reverse transcribed to cDNA. The *PoFAR2* gene sequence (Gene ID: Pos.gene.78836) was retrieved from the tree peony genome (https://ftp.cngb.org/pub/CNSA/data5/CNA0050666). Primers were designed based on this sequence (Table S3), and PCR amplification was performed using high-fidelity K5 DNA polymerase. The PCR products were purified, ligated into the pMD18-T vector, and transformed into *E. coli*. Positive clones were selected and sequence to obtain the full-length *PoFAR2* gene.

### Subcellular localization

The *PoFAR2* open reading frame (without a stop codon) was cloned into the *BamHI* site of the pCAMBIA2300-GFP vector to generate a 35S–*PoFAR2*–GFP fusion construct. After sequencing confirmation, the recombinant plasmid was transformed into *Agrobacterium tumefaciens* GV3101 and introduced into tobacco leaves. Following 2–3 days of dark incubation, fluorescence signals were observed using a laser scanning confocal microscope.

### Promoter activity

A promoter expression vector (0390-35S-GUS) was constructed via homologous recombination, with primer sequences listed in Table S3. Transient transformation of 6-week-old tobacco leaves was performed using vacuum infiltration. A GUS vector without a promoter served as the negative control, while a CaMV35S promoter-driven GUS vector was the positive control. After 1 day of incubation under normal light, GUS staining was performed, followed by decolorization with 70% ethanol, and the staining results were observed.

#### VIGS system Instantaneous transformation assay

A 300-bp *PoFAR2*-specific fragment was inserted into the pTRV2 vector. The pTRV2 plasmid, pTRV2-*PoFAR2* plasmid, and pTRV1 vector were transformed into Agrobacterium tumefaciens GV3101. Bacterial suspensions were adjusted to OD<sub>600</sub> = 0.8–1.0. Two mixtures were prepared: TRV1:TRV2 (1:1 ratio) and TRV1:TRV2-*PoFAR2*, followed by 4-h dark incubation. Tree peony flower buds at the balloon stage were selected for apical injection with bacterial suspensions. Pollen was collected at 3 days post-anthesis. RNA extraction and expression analysis were performed to measure silencing efficiency.

### Statistical analysis

Statistical analyses were performed to evaluate the effects of treatments on seed setting rate, pollen deposition and differential gene expression. Independent-samples t-tests compared fruit yield parameters, endogenous hormone levels, gene relative expression level, pollen abnormal rate and pollen viability. Homogeneity of variances was assessed using Levene’s test, and the one-way ANOVA with LSD *post hoc* test was used for group comparisons. For heterogeneity data, the nonparametric Kruskal–Wallis test followed by Bonferroni correction was applied. Significance level were set *P* < 0.05 and *P* < 0.01.

## Supplementary Material

Web_Material_uhaf224

## Data Availability

The data underlying this article are available in the manuscript and in its online supplementary material.
