# Supplementary material for: Bee-mediated pollination enhances fruit set and seed yield in Paeonia ostii ‘Fengdan’: insights into physiological and molecular mechanisms
Source: Hortic Res. 2025 Nov 1;12(11):uhaf224. doi: 10.1093/hr/uhaf224 (PMC12577852; doi:10.1093/hr/uhaf224)
Supplement: Web_Material_uhaf224 [file web_material_uhaf224.zip › Supplementary table.docx]

Table S1 Statistical table of sequencing data

| Sample | Clean reads | Clean bases | GC Content | %≥Q30 |
| --- | --- | --- | --- | --- |
| NP_0-1_ | 29208456 | 8671795354 | 44.67 | 94.39 |
| NP_0-2_ | 22965785 | 6788502900 | 44.72 | 94.37 |
| NP_0-3_ | 24662096 | 7338142676 | 45.00 | 94.04 |
| BP_1-1_ | 21537861 | 6389306932 | 44.40 | 94.03 |
| BP_1-2_ | 20150014 | 5980343282 | 44.30 | 94.19 |
| BP_1-3_ | 24585315 | 7287298446 | 44.62 | 94.29 |
| BP_2-1_ | 20217789 | 5998028892 | 44.57 | 94.26 |
| BP_2-2_ | 23200010 | 6893055204 | 44.67 | 93.75 |
| BP_2-3_ | 20584759 | 6102910390 | 44.34 | 94.44 |
| BP_4-1_ | 23707404 | 7041675990 | 44.94 | 94.26 |
| BP_4-2_ | 28211120 | 8383946926 | 44.87 | 94.22 |
| BP_4-3_ | 21031608 | 6251899060 | 44.38 | 93.76 |
| BP_8-1_ | 21027354 | 6257633994 | 44.54 | 93.92 |
| BP_8-2_ | 20639346 | 6130796162 | 44.59 | 93.81 |
| BP_8-3_ | 22968247 | 6807447384 | 44.99 | 93.9 |
| BP_24-1_ | 24050503 | 7139938082 | 45.15 | 94.28 |
| BP_24-2_ | 21765400 | 6459923538 | 45.17 | 93.79 |
| BP_24-3_ | 21619083 | 6429595452 | 44.79 | 94.26 |
| BP_48-1_ | 28311831 | 8408621860 | 44.84 | 93.55 |
| BP_48-2_ | 29205201 | 8658878944 | 44.72 | 94.05 |
| BP_48-3_ | 21998986 | 6517552828 | 44.52 | 94.07 |
| BP_72-1_ | 26959895 | 7997889434 | 44.88 | 93.89 |
| BP_72-2_ | 27650177 | 8222346040 | 44.51 | 93.87 |
| BP_72-3_ | 20028745 | 5951015984 | 44.74 | 93.36 |
| SP_1-1_ | 23575952 | 6997363568 | 44.56 | 93.61 |
| SP_1-2_ | 22726952 | 6737348064 | 44.97 | 93.82 |
| SP_1-3_ | 23743210 | 7066726472 | 44.44 | 93.64 |
| SP_2-1_ | 20925190 | 6231597458 | 44.62 | 93.31 |
| SP_2-2_ | 20798034 | 6162036354 | 44.88 | 93.77 |
| SP_2-3_ | 24401860 | 7245727056 | 44.33 | 93.5 |
| SP_4-1_ | 21461387 | 6387778552 | 44.33 | 93.4 |
| SP_4-2_ | 19702958 | 5858109020 | 44.51 | 93.11 |
| SP_4-3_ | 19936691 | 5933051970 | 44.66 | 93.17 |
| SP_8-1_ | 24885001 | 7406194118 | 45.08 | 93.19 |
| SP_8-2_ | 23093525 | 6909753490 | 44.83 | 92.38 |
| SP_8-3_ | 21372882 | 6349410884 | 44.94 | 93.56 |
| SP_24-1_ | 22154993 | 6592183096 | 44.92 | 93.46 |
| SP_24-2_ | 19933993 | 5905228606 | 44.58 | 94.12 |
| SP_24-3_ | 22481185 | 6670464662 | 45.24 | 93.89 |
| SP_48-1_ | 19759961 | 5873267634 | 44.56 | 93.51 |
| SP_48-2_ | 21521718 | 6399173858 | 44.46 | 93.34 |
| SP_48-3_ | 20853622 | 6198170876 | 44.46 | 93.87 |
| SP_72-1_ | 23870742 | 7089238122 | 44.93 | 92.84 |
| SP_72-2_ | 24824452 | 7362402698 | 44.60 | 93.59 |
| SP_72-3_ | 21022918 | 6236001030 | 44.32 | 93.69 |

Table S2 The sequencing data were compared with the reference genome

| Sample | Mapped rate | Sample | Mapped rate |
| --- | --- | --- | --- |
| NP_0-1_ | 84.67% | - | - |
| NP_0-2_ | 84.67% | - | - |
| NP_0-3_ | 79.17% | - | - |
| BP_1-1_ | 82.78% | SP_1-1_ | 84.67% |
| BP_1-2_ | 84.36% | SP_1-2_ | 84.25% |
| BP_1-3_ | 81.85% | SP_1-3_ | 84.68% |
| BP_2-1_ | 84.23% | SP_2-1_ | 85.14% |
| BP_2-2_ | 84.59% | SP_2-2_ | 85.19% |
| BP_2-3_ | 82.77% | SP_2-3_ | 85.75% |
| BP_4-1_ | 84.93% | SP_4-1_ | 84.11% |
| BP_4-2_ | 85.03% | SP_4-2_ | 84.39% |
| BP_4-3_ | 84.75% | SP_4-3_ | 85.07% |
| BP_8-1_ | 83.26% | SP_8-1_ | 83.69% |
| BP_8-2_ | 84.41% | SP_8-2_ | 84.71% |
| BP_8-3_ | 84.71% | SP_8-3_ | 84.89% |
| BP_24-1_ | 84.98% | SP_24-1_ | 85.40% |
| BP_24-2_ | 85.16% | SP_24-2_ | 85.05% |
| BP_24-3_ | 85.39% | SP_24-3_ | 85.34% |
| BP_48-1_ | 84.26% | SP_48-1_ | 84.11% |
| BP_48-2_ | 84.55% | SP_48-2_ | 84.48% |
| BP_48-3_ | 84.41% | SP_48-3_ | 84.58% |
| BP_72-1_ | 84.81% | SP_72-1_ | 83.35% |
| BP_72-2_ | 84.57% | SP_72-2_ | 84.85% |
| BP_72-3_ | 84.55% | SP_72-3_ | 84.60% |

Table S3 Sequence of primers

| Primer name | Primer sequences（5'-3'） |
| --- | --- |
| *PoFAR2*-OE-F | ATGGTTGTGAATGCCACAATG |
| *PoFAR2*-OE-R | TCACCCAGACGCGAATCT |
| Q-*PoFAR2*-OE-F | GAAATGTTGGGAGACCAGGAC |
| Q-*PoFAR2*-OE-R | GTCTCCATTGTCCCGCTTT |
| *PoFAR2*-2300-F | ACAGGGTACCCGGGGATCCATGGTTGTGAATGCCACAATG |
| *PoFAR2*-2300-R | GCCCATGTCGACTCTAGACCCAGACGCGAATCT |
| *PoFAR2*-GUS-F | TGGGCCCGGCGCGCCAAGCTTATCACACACCTAGAACTCATACACT |
| *PoFAR2*-GUS-R | GGTGGACTCCTCTTAGAATTCGAGTCAAATTGCCACATAGACTCT |
