# Supplementary figures and images for: Bee-mediated pollination enhances fruit set and seed yield in Paeonia ostii ‘Fengdan’: insights into physiological and molecular mechanisms
Source: Hortic Res. 2025 Nov 1;12(11):uhaf224. doi: 10.1093/hr/uhaf224 (PMC12577852; doi:10.1093/hr/uhaf224)

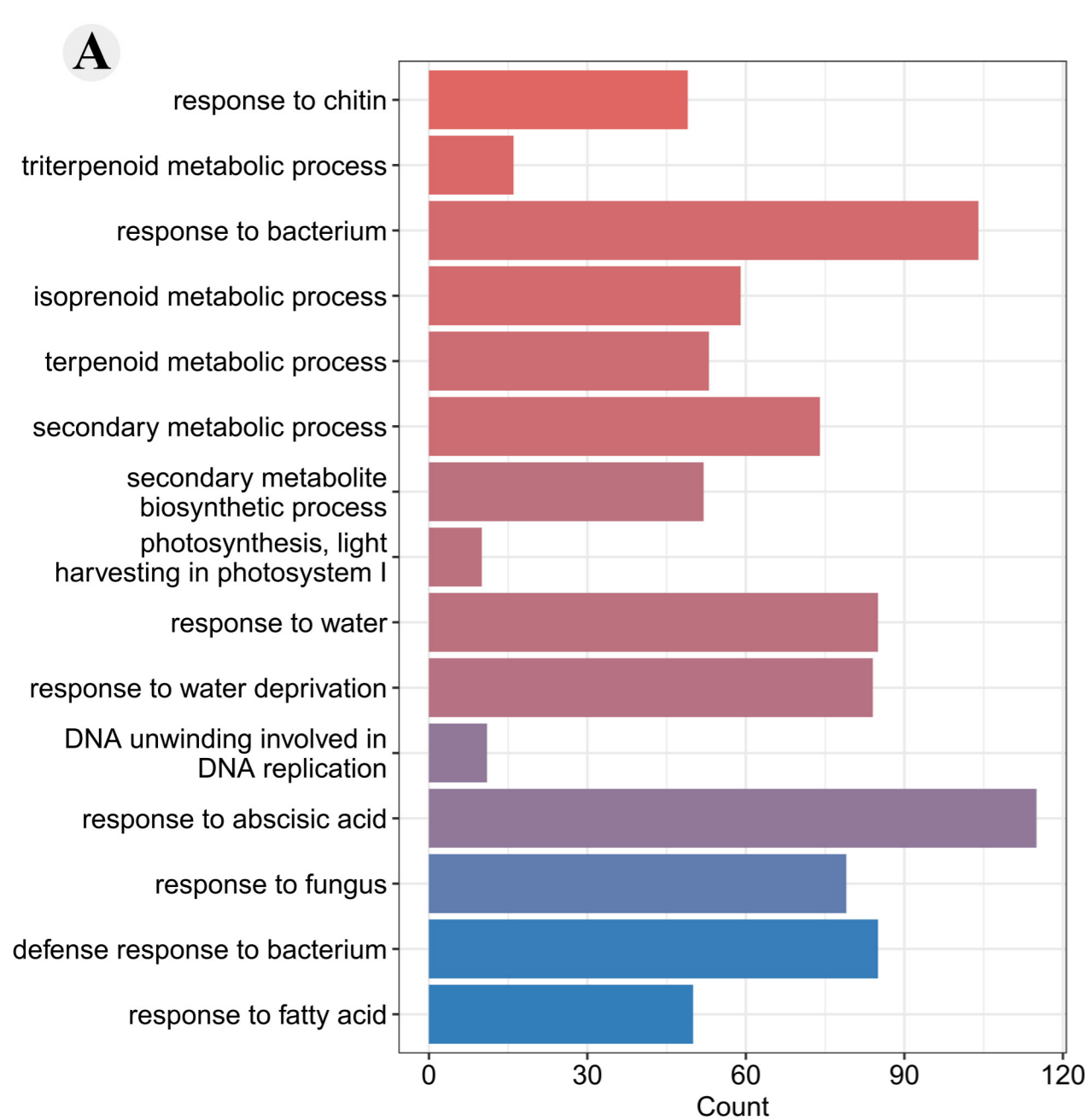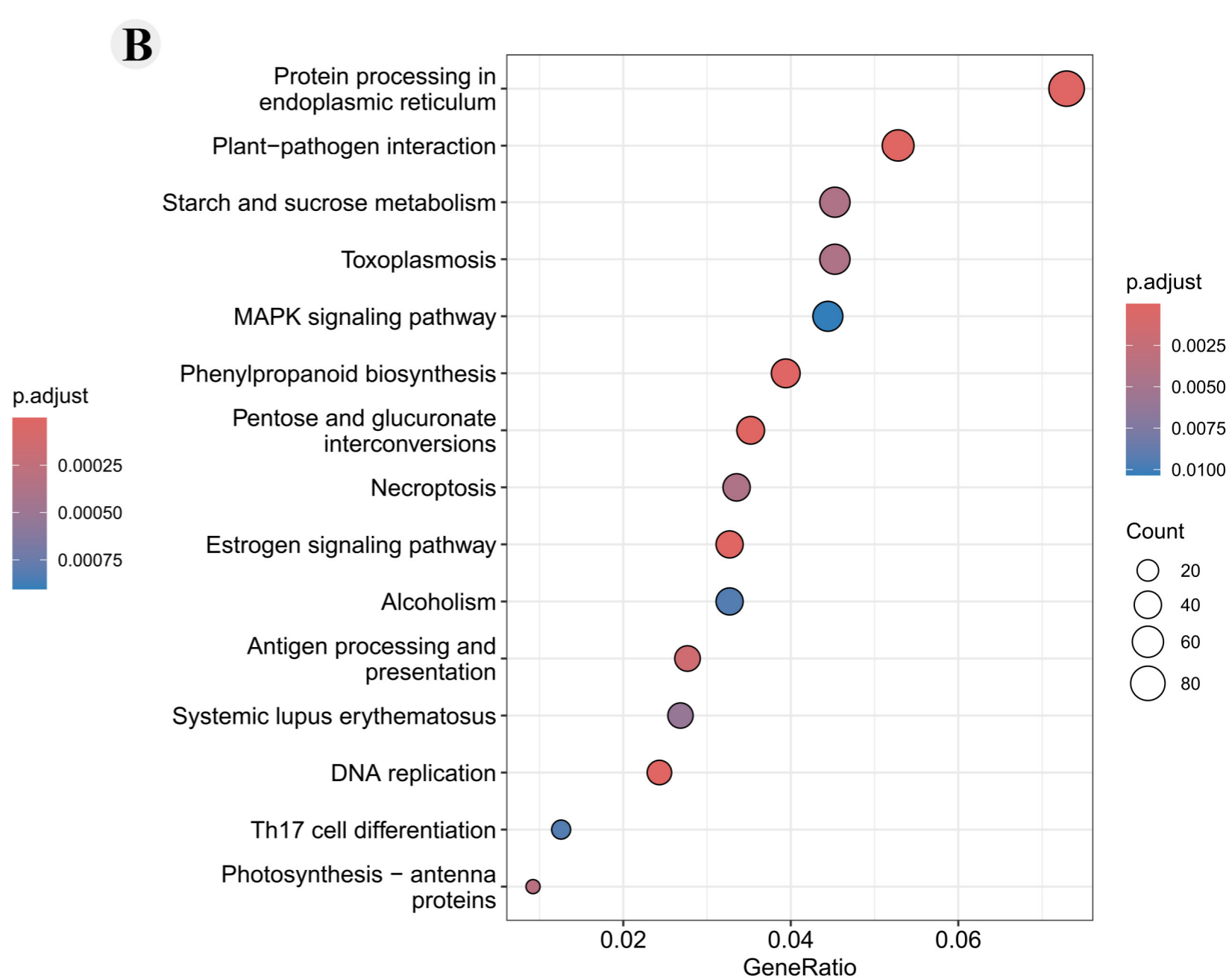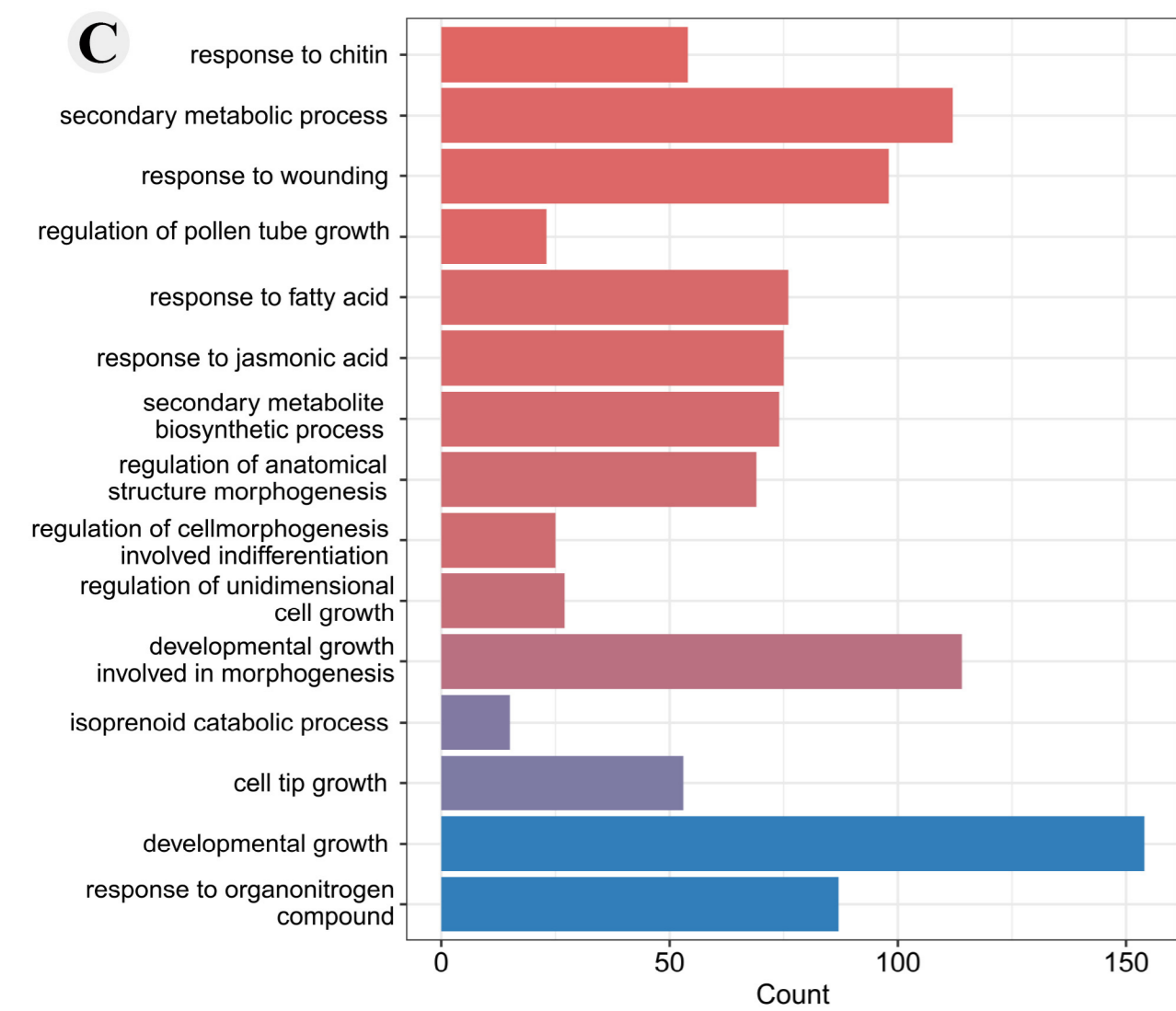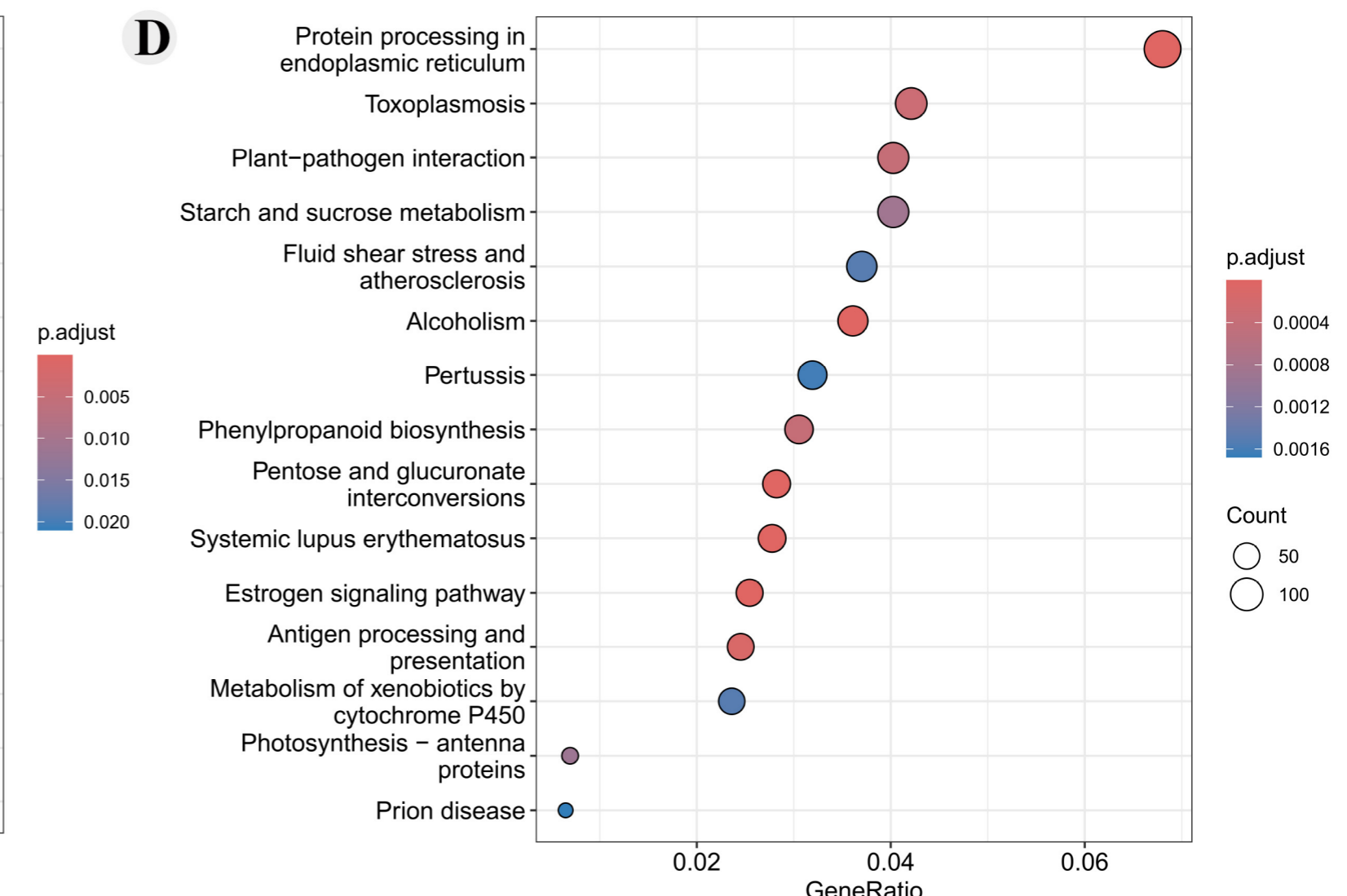

Supplement: Web_Material_uhaf224 [file web_material_uhaf224.zip › Fig. S1.pdf]
